# Simple descriptive missing data indicators in longitudinal studies with attrition, intermittent missing data and a high number of follow-ups

**DOI:** 10.1186/s13104-018-3228-6

**Published:** 2018-02-13

**Authors:** Morten Wærsted, Taran Svenssen Børnick, Jos W. R. Twisk, Kaj Bo Veiersted

**Affiliations:** 1 Department of Work Psychology and Physiology, National Institute of Occupational Health, PO box 8149 Dep, 0033 Oslo, Norway; 2 Department of Epidemiology and Biostatistics, VU Medical Center, De Boelelaan 1089a, 1081 HV Amsterdam, The Netherlands; 3 EMGO Institute for Health and Care Research, VU Medical Center, De Boelelaan 1089a, 1081 HV Amsterdam, The Netherlands

**Keywords:** Longitudinal study, Missing data, Wave nonresponse, Patterns of missingness, Missing data indicators, Attrition

## Abstract

**Objective:**

Missing data in longitudinal studies may constitute a source of bias. We suggest three simple missing data indicators for the initial phase of getting an overview of the missingness pattern in a dataset with a high number of follow-ups. Possible use of the indicators is exemplified in two datasets allowing wave nonresponse; a Norwegian dataset of 420 subjects examined at 21 occasions during 6.5 years and a Dutch dataset of 350 subjects with ten repeated measurements over a period of 35 years.

**Results:**

The indicators *Last response* (the timing of last response), *Retention* (the number of responded follow-ups), and *Dispersion* (the evenness of the distribution of responses) are introduced. The proposed indicators reveal different aspects of the missing data pattern, and may give the researcher a better insight into the pattern of missingness in a study with several follow-ups, as a starting point for analyzing possible bias. Although the indicators are positively correlated to each other, potential predictors of missingness can have a different relationship with different indicators leading to a better understanding of the missing data mechanism in longitudinal studies. These indictors may be useful descriptive tools when starting to look into a longitudinal dataset with many follow-ups.

**Electronic supplementary material:**

The online version of this article (10.1186/s13104-018-3228-6) contains supplementary material, which is available to authorized users.

## Introduction

A longitudinal study with a high number of follow-ups provides a unique opportunity to evaluate individual development over time, but it also implies many challenges. Participants may be present for some waves of data collection and missing for others (*wave nonresponse*). The pattern of missingness may be *monotone* (the subject drops permanently out of the study, often referred to as attrition or dropout), *intermittent* (missing observations between the observed) or *mixed* (an intermittent pattern followed by monotone missingness) [1–6].

Missing data makes standard analyses more difficult or inappropriate to implement, gives loss of efficiency, and under certain circumstances introduces bias [5, 7]. Advanced statistical methods are available in standard statistical software and increase efficiency by using all data collected [8–10]. However, applying these advanced techniques may also introduce bias and requires high statistical skill to avoid pitfalls [9]. For a proper interpretation of study results, it may be important to investigate whether people with different missing data patterns differ from each other in other characteristics. Most literature on missing data has focused on monotone missing data [11], where a common method is to dichotomize participants from baseline to: (1) *Participants who prematurely dropped out*, and (2) *Participants who answered all follow*-*ups* [12]. In datasets with many follow-ups and different patterns of missingness the analysis becomes more complicated [6]. In a study with three follow-ups Ware and co-workers [13] introduced four categories of response patterns: *Always responders* (no missing), *Leavers* (responding to one or two follow-ups, but not the last one), *Returners* (missing one or two follow-ups, but not the last one), and *Never responders* (responding only at baseline). However, these categories are less applicable when the number of follow-ups is large.

This paper proposes three simple descriptive missing data indicators to characterize individual patterns that may arise in a longitudinal study with many follow-ups. These indicators may be a helpful additional tool in the initial phase of getting an overview of the missingness patterns in a dataset. The first indicator is the timing of the last response to follow-up, independent of a participant’s pattern of missingness before the last response. The second indicator is the amount of data each participant contributes with, i.e. the number of follow-ups each participant responded to. The third indicator concerns the evenness of the distribution of responses throughout the whole follow-up period. Together these three simple indicators may be helpful in evaluating the patterns of missing data which is a crucial issue in order to have an idea about the external validity of study results. The aim of this paper is to stress the importance of evaluating the patterns of missing data and this paper provides a few relatively simple tools to do so. As far as we know, the way of addressing this issue and the three proposed descriptive indicators are new inventions of this paper. In order to exemplify the calculation and possible use of these indicators, datasets are obtained from two previously published studies with a longitudinal design and a high number of follow-ups. One dataset comes from a Norwegian study following technical school students through their apprenticeship period and into working life [14]. The other dataset comes from a Dutch study with a follow-up period of 35 years from the age of 12–14 [15].

## Main text

### Methods

#### Sample datasets

The first dataset is drawn from a Norwegian longitudinal study with 20 follow-ups over six and a half years [14], including 420 technical school students (mean age 17.5 years at baseline). Throughout the follow-up period, the participants were allowed to skip one or more follow-ups without being excluded from further participation. We selected four independent variables measured at baseline and known from other studies to have a potential to influence dropout, in addition to the baseline value of the main outcome variable of the original study. These five variables (*Gender*, *Parents’ country of origin, Smoking*, *Self*-*reported health* and *Neck and shoulder pain last 4* *weeks*) were all dichotomized (see the left column of Table 1 in “Results” section). *Self*-*reported health* was rated with a simple question—*How is your health now?* collected from ‘Health Behavior among pupils’, a World Health Organization survey [16]. *Neck and shoulder pain last 4* *weeks* was measured by an index capturing both intensity and duration [14, 17]. More detailed information about this cohort is given elsewhere [14].

The second dataset is drawn from the Amsterdam Growth and Health Longitudinal Study with 10 follow-ups over 35 years [15]. The study started in 1976 with more than 600 boys and girls aged between 12 and 14 years of age. The sample dataset includes the 350 subjects that were invited to all follow-ups. We selected the following variables as potential indicators for missing: *Gender, Biological age, Social desirability, Social inadequacy* and *Physical fitness*. *Biological age* was estimated as skeletal age by radiographs of the left hand and wrist. *Social desirability* was measured with the Achievement Motivation Test [18], while *Social inadequacy* was measured with the Dutch Personality Inventory [19]. *Physical fitness* was assessed by measuring maximal oxygen uptake (VO_2_max) by running on a treadmill [20].

#### Descriptive missing data indicators

The *Last response indicator* uses the timing of the last follow-up measurement that was responded to, giving a score of zero for only responding at baseline and the maximum score of 100 for responding at the last follow-up.

The *Retention indicator* reflects the exact number of measurements responded to relative to the total number of measurements in the study protocol, giving a score of zero for only taking part in the baseline measurement and the maximal score of 100 for taking part in all follow-up measurements in the study.

The *Dispersion indicator* quantifies to what extent the attended follow-up measurements are spread evenly throughout the follow-up period. The calculation of the *Dispersion indicator* implies for a given number of attended follow-up measurements, defining a theoretical optimal number of missed measurements between two consecutive attended measurements, including the period from the last measurement until the end of the study, which will spread the responded measurements evenly throughout the study period. This theoretical optimal number will not be an integer, and is only used for calculation purposes. For each answered follow-up the deviation from optimal number of missed measurements until the next attended measurement (or end of study) is squared to give weight to large deviations and summed up for all attended measurements of the subjects. The sum is normalized to a score between zero and 100 and turned to give increasing score on the *Dispersion indicator* with increasing dispersion of the attended measurements. The exact calculation steps needed to obtain the *Dispersion indicator* are listed in Additional file 1. This calculation is less straight forward than the calculation of the first two indicators, and will most conveniently be done with a custom-made program (see an example in Additional file 2).

**Fig. 1:**
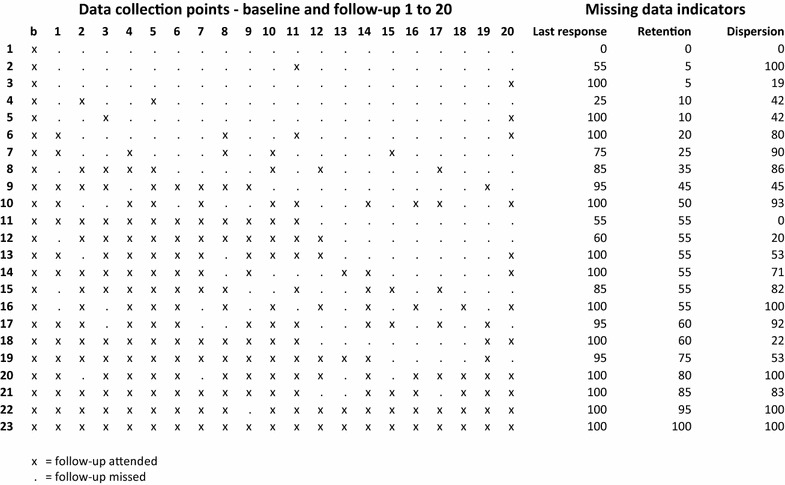
Examples on response patterns and missing data indicator scores. Each line represents the response pattern of one subject, listed according to increasing scores on the Retention indicator. The scores are standardized between 0 and 100. A subject responding only at baseline will get a score of zero on all indicators (subject 1); while a subject responding to all follow-ups will get a score of 100 on all indicators (subject 23). The 23 response patterns illustrated in this figure are all drawn from observed response patterns among the 420 subjects of the Norwegian sample dataset, with the exception for subject 16 who is added for illustrative purposes. See main text for more detailed description of the three missing data indicators

Figure 1 gives concrete examples of the proposed missing data indicators calculated for selected subjects drawn from the Norwegian dataset.

#### Statistical analysis

Mann–Whitney U tests and Spearman correlation coefficients (IBM SPSS version 21.0) were used to relate variables potentially related to missingness to the missing data indicators. Non-parametric methods were used because the missing data indicators could not be assumed to be symmetrically distributed. In addition, Spearman correlation coefficients were calculated to assess the correlations between the missing data indicators. A p value < .05 was considered significant.

### Results

**Fig. 2:**
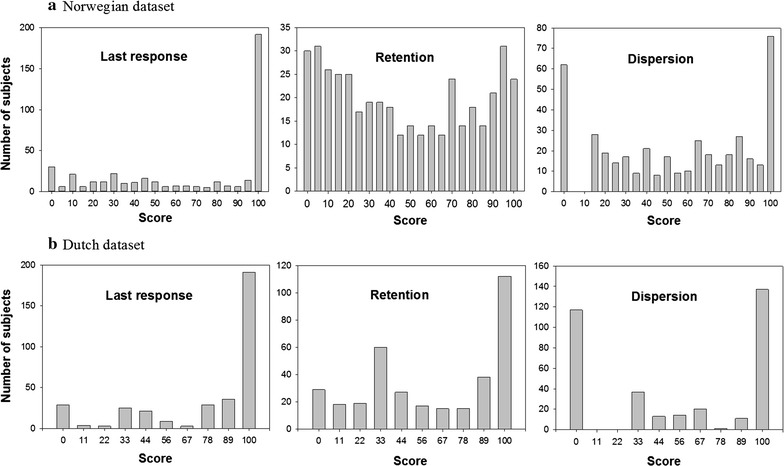
Distribution of the three missing data indicators. The scores of the 420 participants of the Norwegian dataset (**a**) and the 350 participants of the Dutch dataset (**b**). In each dataset the scores of the Dispersion indicator are for comparison put in bins centered on the same values as the possible values of the other two indicators. The pairwise Spearman correlation coefficients for the Last response (LR), Retention (Ret) and Dispersion (Dis) indicators were LR-Ret 0.82, LR-Dis 0.79, Ret-Dis 0.76 (Norwegian dataset) and LR-Ret 0.68, LR-Dis 0.82, Ret-Dis 0.74 (Dutch dataset). All these correlation coefficients were highly significant (p < .001)

The distribution of the three missing data indicators is shown in Fig. 2.

In the Norwegian dataset (Table 1), smokers compared to non-smokers and participants with at least one parent of non-western origin had lower scores for all the missing data indicators. Men scored lower than women on the *Retention indicator*. Neither *Self*-*reported health* nor *Neck and shoulder pain last 4* *weeks* differed between the categories for any of the indicators.

In the Dutch dataset (Table 1), gender was not related to any of the missing data indicators. Biological age and social desirability were inversely related to the *Last response indicator*. Physical fitness was positively associated with the missing data indicators *Last response* and *Dispersion*. Although in general, the observed relationships were relatively small.

**Table 1:** Bivariate relationships in the two sample datasets

Missing data indicators	Last response		Retention		Dispersion	
	Median (percentiles 25–75%)	p	Median (percentiles 25–75%)	p	Median (percentiles 25–75%)	p
*Norwegian dataset (n = 420)*						
Gender						
Men (n = 153)	85 (25–100)	.43	30 (10–75)	.016	65 (18–88)	1.0
Woman (n = 267)	90 (35–100)		50 (20–80)		50 (22–89)	
Parents’ country of origin						
Both western (n = 368)	95 (35–100)	.015	50 (20–80)	< .001	64 (21–90)	.050
One or both non-western (n = 52)	53 (20–100)		20 (5–44)		43 (5–78)	
Smoking (n = 419)						
Never/former/sometimes (n = 279)	100 (35–100)	.005	55 (20–83)	.001	67 (22–92)	.017
Every day (n = 140)	65 (30–100)		35 (10–60)		50 (19–76)	
Self–reported health						
Good/very good (n = 306)	95 (30–100)	.41	40 (15–80)	.84	54 (19–88)	.71
Not quite good/poor (n = 114)	78 (34–100)		45 (20–80)		51 (26–88)	
Neck and shoulder pain last 4 weeks						
No (0–1) (n = 279)	95 (30–100)	.73	40 (15–80)	.99	58 (19–90)	.92
Yes (2–12) (n = 141)	80 (35–100		50 (18–75)		64 (28–85)	
*Dutch dataset (n = 350)*						
Gender						
Men (n = 169)	100 (56–100)	.93	56 (33–100)	.25	50 (0–100)	.42
Woman (n = 267)	100 (56–100)		67 (33–100)		62 (0–100)	

	Spearman correlations	p	Spearman correlations	p	Spearman correlations	p
Biological age	− 0.140	.009	− 0.006	.92	− 0.078	.15
Social desirability	− 0.143	.008	− 0.088	.10	− 0.094	.08
Social inadequacy	− 0.104	.054	− 0.082	.13	− 0.070	.19
Physical fitness	0.174	.001	0.051	.35	0.131	.016

### Discussion

In order to highlight different individual patterns in missingness, we have introduced three missing data indicators. We propose these indicators as descriptive tools in an early stage of evaluating a dataset with a high number of follow-ups and wave nonresponse with monotone, intermittent or mixed missingness. By using these indicators, the researcher may observe interesting patterns of missingness that may be overlooked in ordinary analysis and that may be potential sources of selection bias. The three indicators were constructed to catch in a simple manner three different aspects of an individual response pattern in a longitudinal study where all participants participate at baseline.

The *Last response indicator* gives a simple measure of how long into the study the subject is observed. This may be the most important aspects to evaluate for research questions where a long follow-up is crucial, and can be viewed as an indicator of dropout from a study.

The *Retention indicator* reflects the total amount of data each participant contributes. For many purposes, a high response rate may be regarded as the most important aspect.

The *Dispersion indicator* captures to what extent the data from a subject cover a large part of the study period relative to the number of data collections attended to. The argument for constructing this indicator is the value of having subjects contributing throughout a study period. Thus, in most studies an even distribution of attended data collections may be viewed as optimal, given the number of data collections where the subject contributed. For several research questions this quality will add to the validity of the data contributed from the subject. Constructing an indicator for this aspect, however, is not as straight forward as for the two other indicators. We chose to score the evenness of the distribution of responses given the total number of responses, so that for every number of responses both a maximal (100) and a minimal (0) score was possible. Simpler logics for calculating a *Dispersion indicator* were contemplated; however, the logic presented in this paper gave the best reflection of the dispersion of the responses, and in the best way supplemented the other two indicators in addressing the different aspects of the missingness patterns. When a subject has very high or very low attendance, the *Dispersion indicator* is not so informative. One possible option can be not to include subjects with very high or very low scores on the *Retention indicator* when evaluating the *Dispersion indicator*.

The missing data indicators introduced in this paper, put numbers on three aspects of the patterns of missed follow-ups that may arise in a study with intermittent missing and a high number of follow-ups. The intention is to provide a tool that may help a researcher in getting an initial overview of the missingness in a dataset, as a supplement to the well-established methods to evaluate and handle missing data. The indicators are not linked to modelling or outcome, and are not constructed to give values that may be used to decide on the acceptability of the data or to decide on which methods to use to handle the missingness. However, a better understanding of the missingness patterns in a dataset will be of value when looking for possible sources of bias and deciding on further steps in the data analysis. In this respect, some researchers may find the three proposed indicators helpful, depending on the way they prefer to get an overview of their datasets.

## Limitations

It should be realized that the three missing data indicators can only be calculated when the total number of follow-up measurements is known. In most longitudinal studies, this would probably be the case. However, when the follow-up time points are unplanned, the three missing data indicators are not well defined. Besides that, a prerequisite to use the three proposed indicators is that a study protocol allows intermittent missingness or mixed missingness, which is mostly the case in long-term follow-up cohort studies. The more follow-up measurements, the more informative the three indicators will be. However, also with four or five follow-up measurements the first two indicators can be used, while more follow-ups are needed for the *Dispersion indicator* to be interesting. In our sample datasets, all the missing data indicators show a relatively strong interrelationship. This is partly a consequence of the way the indicators are constructed, but does not mean that they convey identical information. Anyhow, the strength of this relationship might differ between datasets, and more interestingly, the associations between the missing data indicators and particular variables in the dataset will vary as illustrated in Table 1.

## Additional files

**Additional file 1.** The file contains a table listing the calculation steps needed to obtain the *Dispersion indicator*.

**Additional file 2.** The file gives an example of a Phyton program to calculate the three missing data indicators.

## Abbreviation

VO_2_max: maximal oxygen uptake
