# Supplementary material for: Simple descriptive missing data indicators in longitudinal studies with attrition, intermittent missing data and a high number of follow-ups
Source: BMC Res Notes. 2018 Feb 13;11:123. doi: 10.1186/s13104-018-3228-6 (PMC5809924; doi:10.1186/s13104-018-3228-6)
Supplement: Supplementary file 1 — Additional file 1. The file contains a table listing the calculation steps needed to obtain the Dispersion indicator. [file 13104_2018_3228_MOESM1_ESM.docx]

__________________________________________________

**Additional file 1. Calculating the Dispersion Indicator**

Preparing the dataset

1. Assign a variable for each scheduled data collection points (baseline and follow-ups) indicating **yes** or **no** on whether the subject has attended or not. All subjects will have **yes** for baseline.
2. Subjects that have attended all data collection points shall have a dispersion indicator score of **100**.
3. Subjects attending only baseline shall have a dispersion indicator score of **zero**.
4. For all other subjects do the calculation steps 6-15.
5. Before doing the calculations add an extra “data collection point” after the final follow-up of the project period. Regard this fictitious time point as real in all the following calculations and assign the value for **no** for all subjects.

Calculating the individual dispersion indicator scores

1. Count number of attended data collection points (including baseline) = **A**
2. Count number of unattended follow-ups (including the fictitious follow-up) = **B**
3. Calculate: **C = B / A**
4. For each attended data collection point (baseline and follow-ups), count the number of unattended follow-ups until next attended follow-up = **D**. For the last attended follow-up count number of data collection points until the end of the project period (including the fictitious time point).
5. Calculate: **E = (D – C)^2^**
6. **Sum E** for all attended data collection points of that subject **= F**
7. Calculate the lowest possible value of **F** for the actual number of **A**, by doing calculation steps 9-11 when **D** is differing with a maximum of one **= F_min_**
8. Calculate the highest possible value of **F** for the actual number of **A**, by doing calculation steps 9-11 when all attended follow-ups come consecutively after baseline followed by all the missed follow-ups **= F_max_**
9. Calculate: **G = (F – F_min_) / (F_max_ – F_min_) x 100**
10. Calculate the **Dispersion indicator score = 100 – G**

An example of a custom-made Phyton program (version 2.7) calculating all three missing data indicators is included as an additional file [see Additional file 2].

__________________________________________________
